# Theoretical Comparison of Different Envelope Elimination and Restoration Transmitter PWM Modulator Configurations to Expand the Possible Antenna Mismatch

**DOI:** 10.3390/s23239466

**Published:** 2023-11-28

**Authors:** Dang Canh Nguyen, Victor N. Gromorushkin, Oleg Varlamov

**Affiliations:** Department of Radio Equipment and Circuitry, Moscow Technical University of Communication and Informatics, 111024 Moscow, Russia; nguyendangcanh1319951@gmail.com (D.C.N.); grom@mtuci.ru (V.N.G.)

**Keywords:** antenna mismatch, envelope elimination and restoration, envelope path bandwidth, high-efficiency RF power amplifier, LPF, simulation model, switching-mode class D

## Abstract

The main characteristics of high-efficiency switching-mode solid-state power amplifiers with envelope elimination and restoration (EER) methods depend on all their elements. In this article, we study the influence of the types and parameters of the envelope path low-pass filters (LPFs) on the EER transmitter out-of-band emissions. This article presents for the first time an analysis of EER transmitter operation where the output impedance of the PWM modulator is not equal to zero, as usual (with a one-sided loaded LPF), but is matched with the low-pass filter and the load (with a double-sided loaded LPF). Theoretical comparisons of EER transmitters’ out-of-band emissions were carried out with four envelope path LPF configurations (one-sided and double-sided loaded LPFs with a smooth and sharp transition, respectively), for both the nominal load (broadband antenna) and resonant antennas with a limited bandwidth. The analysis showed that for the case of transmitter operation on a resonant antenna with a limited bandwidth, the preferable option was the use of a sixth-order double-sided loaded LPF with a smooth transition. The use of the proposed modulator configuration allowed the transmitter to operate on an antenna with VSWR = 1.07 at the edges of the transmitted signal band with a minimum LPF bandwidth equal to 5.8 bands of the amplified signal. This could significantly expand its application capabilities and allow one to reduce the PWM clock frequency and increase efficiency.

## 1. Introduction

Modern spectrally efficient telecommunication signals used in mobile communication networks [1,2], the Internet of Things, and digital television and radio broadcasting are characterized by a high crest factor (peak to average power ratio), reaching values of 8–10 dB. Traditional methods of linear RF amplification in this case are characterized by low average efficiency—as a rule, about 15%—which does not allow the long-term operation of the subscriber device from one set of batteries and does not provide energy efficiency in high-power applications.

To increase the average efficiency when amplifying signals with a large crest factor, various synthetic amplification methods [3,4] and their combinations [5] have been proposed. Among them, the method of envelope elimination and restoration (EER, or the Kahn method) [4] is the most common and most studied [6]. This method of highly efficient power amplification celebrated its seventieth anniversary last year and is well known to anyone involved in the research and development of high-efficiency power amplifiers or transmitters. Most high-power tube and semiconductor AM broadcast transmitters have used this method since the late 1980s to reduce energy consumption.

Currently, there are more than 220 entries in the IEEE Xplore digital library alone corresponding to a search for “envelope elimination and restoration”. At the same time, in libraries before the Internet era, one can find a number of detailed monographs, for example [7], and even university textbooks [8] for students of telecommunications specialties. Currently, EER power amplifiers are used for digital television and radio broadcasting, as well as for LTE and other applications with a high crest factor.

**Figure 1:**
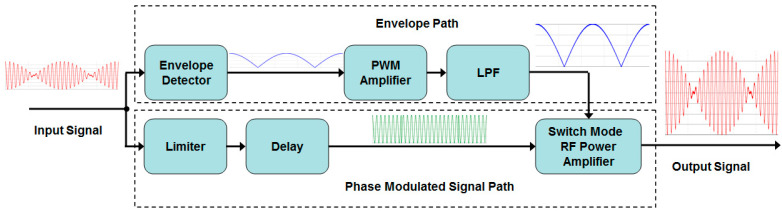
Block diagram of EER technique.

Block diagrams of the EER technique have been described in detail in many works, for example [9], as illustrated in Figure 1.

Powerful RF path operating features [10], including for a mismatched load [11], have been considered in sufficient detail. A number of studies on the features of EER transmitter operation with a mismatched load [12], including a narrow-band (NB) load [13,14], have revealed very stringent requirements for the allowable antenna parameters. Thus, in order to comply with the standards for electromagnetic compatibility (EMC) in terms of out-of-band emissions, for some transmitters it is required to ensure that the voltage standing wave ratio (VSWR) of the NB antenna is no more than 1.05 in the frequency band of the amplified signal.

**Figure 2:**
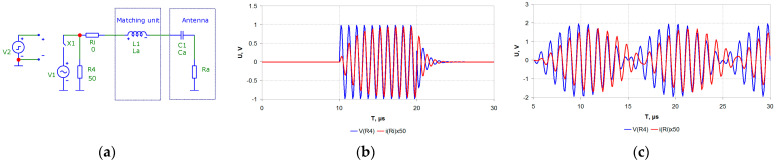
A typical equivalent circuit of an NB antenna and its matching unit (**a**). The results for the impact of a radio pulse (**b**) and a two-tone equal-amplitude signal with a frequency spacing of 100 kHz (**c**) on an NB antenna with a quality factor Qa = 5 and a resonant frequency of 1000 kHz.

These requirements apply to transmitters that use the most common and energy-efficient envelope amplification path with pulse-width modulation (PWM) [15] and are not applicable to other methods of constructing modulation paths. The assumed physical causes of this effect were considered in [16]. A typical equivalent circuit of an NB electrically short antenna (e.g., a short vertical monopole) and its matching unit are shown in Figure 2a [16]. The results for the impact of a radio pulse and a two-tone equal-amplitude signal with a frequency spacing of 100 kHz on an NB antenna with a quality factor Qa = 5 and a resonant frequency of 1000 kHz are shown in Figure 2b,c as an example. The blue line shows the voltage in the antenna, and the red line shows the current in the antenna. At some time intervals, the input voltage and current of the antenna are out-of-phase. That is, the energy stored in the high-Q antenna circuit is fed back to the transmitter. In the case of a linear power amplifier, it is dissipated by the elements that are in active mode. There are no dissipative elements in the switching EER transmitter, and the energy received from the antenna causes parasitic oscillations in the low-pass filter of the modulator, leading to an increase in out-of-band radiation.

It should be noted that the well-known nonlinear load analysis carried out in [17,18] for envelope tracking (ET) PAs is not applicable in the case of EER PAs with switching operating modes for the transistors. Indeed, when the ET PA is in operation, the RF stage transistors operate in an active (linear) mode that is the equivalent of an adjustable resistance between the RF load and the output of the envelope amplifier.

When the HF stage in the EEP PA operates in a switching mode, it practically represents a “short circuit” between the HF load and the output of the envelope amplifier in the low-frequency region. Also, unlike the ET PA, any envelope path mismatch is unacceptable for the EER PA, because this will lead to the significant distortion of the output signal [7].

The experimental confirmation of the permissible NB antenna SWR limitation for an EER transmitter was carried out by the authors of [14]. The search for theoretical literature on this topic did not yield any results, which indicates the relevance and novelty of the research being carried out.

In order to study in detail the causes of this effect and possible ways to minimize its consequences, the authors developed a simulation model to study the operation of switching-mode EER RF power amplifiers for an NB load [19]. As shown in [19], widespread modeling systems such as ADS and MWO do not allow one to directly perform the simulation of this problem. In this article, using the model in [19], an analysis is conducted of the envelope path filter parameters’ influence on the EER transmitter output signal distortion, including on the NB load. The model works using a recorded fragment of a real 10 kHz bandwidth OFDM Digital Radio Mondiale (DRM) signal, and its results can be proportionally scaled. The model is also planned to be used in the future to study reverse intermodulation distortion in EER transmitters.

The low-pass filter (LPF) in the EER transmitter envelope path is designed to suppress the PWM modulator clock frequency and its harmonics. To ensure the required level of clock frequency product suppression, a sixth-order Elliptic (Cauer) LPF with a stopband attenuation no less than 65…75 dB is typically used. The Elliptic (Cauer) LPF structure provides the LPF minimum order with the required PWM clock product suppression. A side effect of the LPF on the transmitter operation is the envelope spectrum limitation, which leads to transmitted signal distortion. In addition, the LPF introduces a significant delay in the envelope signal, which also leads to the distortion of the transmitter output signal. However, in modern transmitters, the delay difference of the envelope and RF components is easily compensated for in the exciter.

This article considers possible options for the implementation of the envelope path LPF and investigates the dependence of the transmitter output signal distortion on the LPF parameters, including when the transmitter is operating on an NB antenna.

The article is organized as follows. The second section contains an overview of the envelope path filter types considered. In the third section, an analysis of the transmitter’s output signal spectral characteristics during its operation for a rated resistive load is carried out. In the fourth section, the relationship between antenna bandwidth and maximum VSWR at the signal band edges is considered. The fifth section presents the results of the analysis of the transmitter’s output signal spectral characteristics when it is working on an NB antenna, as well as studying the dependence of the minimum required envelope path bandwidth on the antenna VSWR value in the signal bandwidth. Recommendations for the selection of the envelope path LPF parameters are set out in the sixth section. Finally, the conclusions are gathered in Section 7.

## 2. Overview of Considered Envelope Path Filter Types

As already noted, a sixth-order LPF with an Elliptic (Cauer) structure is needed to provide the required suppression of PWM clock frequency products. Increasing or decreasing the filter order is impractical, since, in the first case, an unjustified complication of the envelope path and a decrease in its efficiency occur. In the second case, the required suppression of PWM clock frequency products is not provided and, accordingly, the EMC requirements of regulatory documents are not met [20]. Thus, in further analysis we will consider two variants of the sixth-order Elliptic (Cauer) LPF—one-sided loaded (OSL) and double-sided loaded (DSL)—when the source output resistance is equal to zero (R_S_ = 0) or equal to the load resistance of the envelope path (R_S_ = R_L_). The LPF design process can be performed as a standard procedure using tabular coefficients according to [21] depending on the filter order, R_S_ and R_L_ values, allowable passband ripple, required stopband attenuation, and transition region width, followed by calculating the element values relative to the cutoff frequency. In addition, we will consider two options (with R_S_ = 0 and R_S_ = RL) for the optimized LPF with a smooth transition (ST) of the frequency response (FR) from the passband to the stopband. Let us take a closer look at this type of LPF.

**Figure 3:**
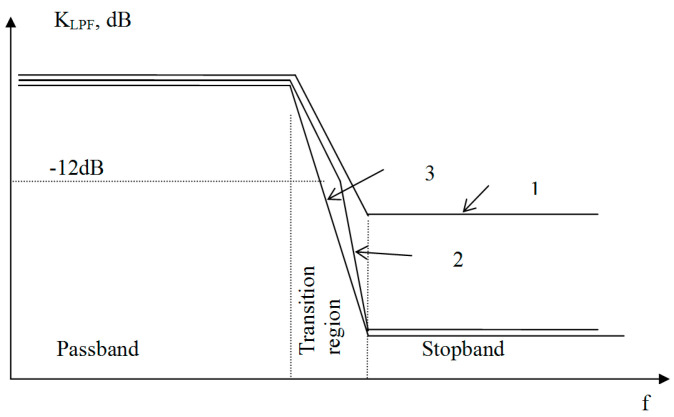
An example of the two ways to implement the FR of an LPF with a low steepness in the initial section of the transition region: 1—LPF with low suppression; 2—LPF with ST; 3—standard LPF with a sharp break in the FR.

As shown by preliminary studies, a sharp break in the FR of the filter at the upper boundary of its passband leads to an increase in the out-of-band radiation of the transmitter in frequency zones that are above and below its central operating frequency at a distance equal to the cutoff frequency of the LPF. There are two ways to remedy this shortcoming. The first is to reduce the calculated low-pass suppression so that the transition region from the passband to the stopband will have a low steepness; however, the necessary suppression of the PWM clock frequency will not be provided. The second is to ensure a small slope for the FR decay in only the initial section of the transition region. Examples of the two ways to implement the FR of the envelope path filter are shown in Figure 3. As preliminary studies have shown, it is sufficient to attenuate the envelope signal by 10…12 dB with a low-steepness transition region so that additional out-of-band transmitter emissions do not occur.

It should be noted that the low-slope LPF in the initial part of the transition region, which will be called the “soft-transition LPF” for brevity, does not have a standard approximation, such as the Elliptic (Cauer) LPF. The synthesis of such filters is carried out by the method of parametric optimization, in which the objective function includes not only the shape of the FR in the initial section of the transition region but also the unevenness of the FR in the passband, as well as the degree of suppression in the stopband.

**Figure 4:**
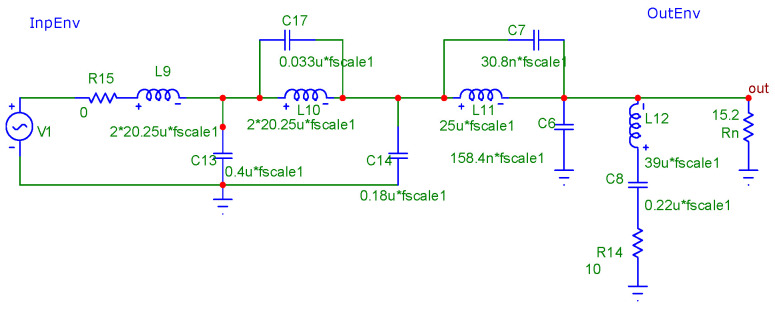
Schematic diagram of the Harris DAX transmitter envelope path LPF (The “*” sign in MicroCap-12 program means multiplication sign).

Let us also consider the available circuits for implementing the envelope path LPF in real transmitters. Regarding circuit diagrams of modern digital transmitters, circuits of J-1000 transmitters from Nautel (Nautel Ltd. Hackett’s Cove, NS, Canada) [22] and transmitters of the DAX series from Harris (currently GatesAir, Inc., Mason, OH/Quincy, IL, USA) [23] are available. The structures of the envelope path LPFs of both transmitters are similar, being sixth-order ladder LPFs with the Elliptic (Cauer) structure. The LPF circuit used in the Harris DAX transmitter envelope amplification path is shown in Figure 4.

Elements L12, C8, and R14, as indicated in the transmitter description, are installed “optionally” and in fact form a damping circuit that reduces the quality factor of spurious resonances in the upper part of the LPF bandwidth when the transmitter is working on an NB antenna.

**Figure 5:**
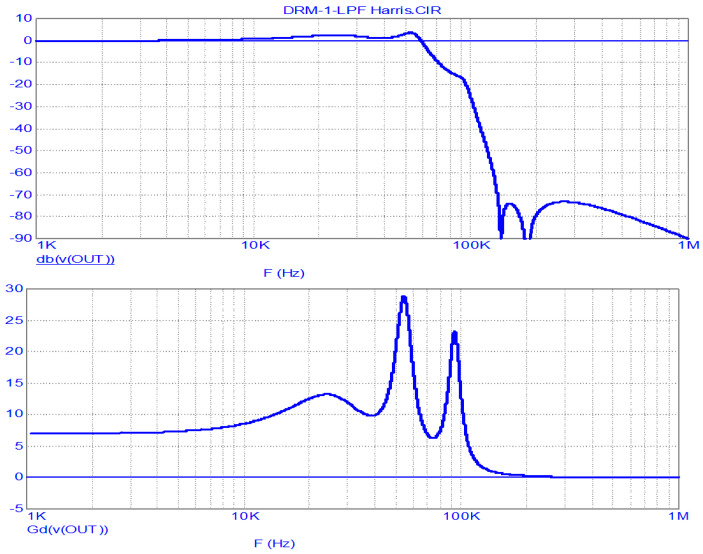
LPF FR (**above**) and GD (**below**) analysis results for the Harris DAX transmitter at R_S_ = 0.

The FR and group delay (GD) analysis results of this LPF, obtained in the MicroCap-12 program for the case of the LPF operating from a voltage generator (R_S_ = R15 = 0) with the optional circuit turned off, are shown in Figure 5.

As can be seen from the graphs, the LPF bandwidth at the level of −3 dB is 62 kHz, and the GD in the 0…10 kHz band (in the envelope’s main energy concentration band) is 7…8.5 µs and has noticeable non-uniformity. The suppression of the PWM clock frequency and its harmonics is no less than 73 dB.

**Figure 6:**
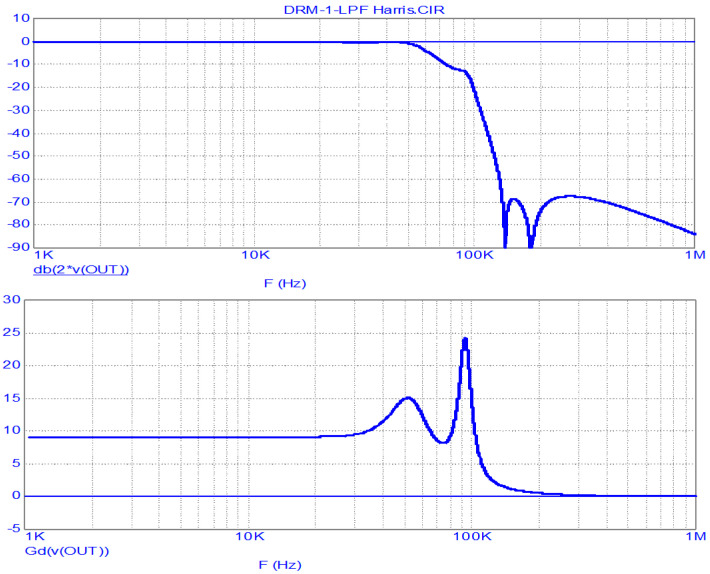
LPF FR (**above**) and GD (**below**) analysis results for the Harris DAX transmitter at R_S_ = R_L_ (matched generator). (The “*” sign in MicroCap-12 program means multiplication sign).

Estimating the given FR and GD, one should note their significant non-uniformity in the passband. Therefore, a similar analysis is carried out on the LPF operating from a matched generator (R_S_ = R_L_ = 15.2 Ω), the results of which are shown in Figure 6.

It should be noted that the FR and GD estimations given in Figure 6 take a classic form. The bandwidth of the LPF at a level of −3 dB is 58 kHz, and the GD in the band 0…20 kHz (in the double band of the envelope’s main energy concentration) is uniform at 9 μs. The suppression of the clock frequency and its harmonics is no less than 67 dB.

Of particular note is the smooth section of the FR transition from the upper frequency of the passband to the stopband, where the FR smoothly decreases to −12 dB. This makes it possible to classify the analyzed filter as an LPF with an ST, which was discussed above.

The analysis of the Nautel transmitter’s envelope path filter FR showed that it has a standard FR with a sharp break, which should lead to an increase in out-of-band emissions. However, it should be noted that digital signal processing is widely used in Nautel transmitters, making it easy to implement an ST in the envelope path FR.

## 3. Analysis of the Transmitter’s Output Signal Spectral Characteristics during Its Operation for a Rated Resistive Load

### 3.1. LPF from Harris DAX Transmitter

**Figure 7:**
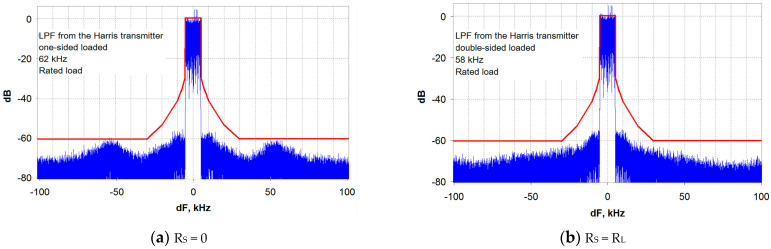
The transmitter output signal spectrum with an LPF from the Harris transmitter: (**a**) for an OSL LPF; (**b**) for a DSL LPF.

Let us now turn to the analysis of the transmitter output signal spectral characteristics with the selected LPF types in the envelope path. The analysis is carried out using the EER transmitter model developed in [19] when operating at a nominal load (broadband antenna). Figure 7 shows the results of the distortion level study for the transmitter’s output signal with the envelope path LPF from the Harris transmitter. The results are given for two variants of the modulator output impedance: zero (R_S_ = 0, Figure 7a) and matched with the load (R_S_ = R_L_, Figure 7b).

As can be seen from Figure 7, at R_S_ = 0, the transmitter output signal spectrum (Blue line) is practically at the limit of the EMC requirements (Red line) [20], and there is no margin left for other causes of distortion. The reason for this is the significant non-uniformity of the LPF FR, highlighted in Figure 5. As noted earlier, the FR correction unit installed in the Harris DAX series transmitter probably compensates for this unevenness, and in practice the situation is somewhat better.

It is a completely different matter when a DSL LPF is operating from a matched generator (with R_S_ = R_L_). In this case, there is a margin for the level of out-of-band emissions of about 6 dB (Figure 7b), which can meet the EMC requirements when the transmitter is operating on an NB antenna.

### 3.2. One-Sided Loaded and Double-Sided Loaded LPFs with a Smooth Transition

**Figure 8:**
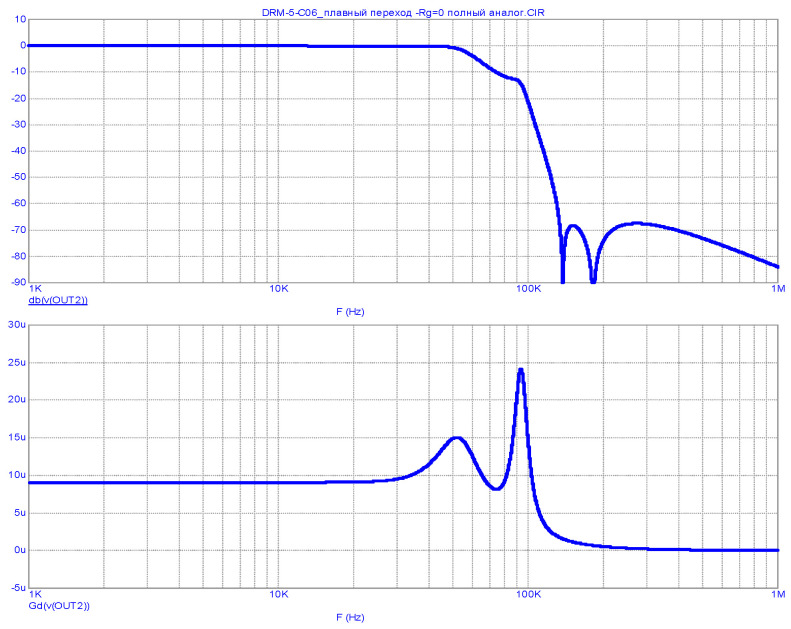
FR (**top**) and GD (**bottom**) of an OSL-optimized LPF with an ST at R_S_ = 0.

Note that in the transmitter model [19], there is no FR correction for the inadequate use of filters. Therefore, for the case of using a modulator with zero output impedance (as in a real transmitter), an OSL LPF with an ST is designed. Its structure is similar to that shown in Figure 4 and differs only in the ratings of the LC elements. Its FR and GD when excited from a voltage source (R_S_ = 0) are shown in Figure 8.

**Figure 9:**
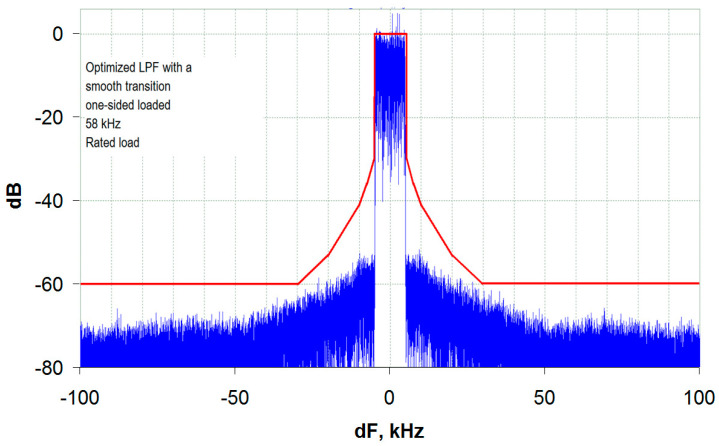
The transmitter output signal spectrum for an optimized OSL LPF with an ST and a bandwidth of 58 kHz (transmitted signal bandwidth of 5.8) at Rg = 0.

As can be seen from Figure 8, the FR and GD of the designed LPF at Rg = 0 are completely identical to the corresponding characteristics of the matched (at R_S_ = R_L_) low-pass filter from the Harris transmitter (see Figure 6). The results of the transmitter output signal spectrum analysis for the model using an optimized OSL LPF are shown in Figure 9.

As can be seen from Figure 9, when operating a transmitter featuring an optimized OSL LPF with an ST, the spectrum margin from the mask is about 6 dB. The nature of the spectrum is almost identical to the spectrum in Figure 7b for a similar DSL filter from a Harris transmitter. The out-of-band emission levels decrease monotonically with increasing detuning from the center frequency.

Based on the simulation, it can be argued that when the transmitter is operating at a nominal load (broadband antenna), it does not matter which LPF is used—OSL or DSL. It is important that the signal source (modulator) output impedance matches that adopted when designing the filter. In this case, there is no need to correct the frequency characteristics of the low-frequency path.

The second important point to be noted is that with a transmitter low-frequency (envelope) path bandwidth of about six bands of the transmitted signal, the out-of-band emissions caused by the envelope spectrum limitation lie 6 dB below the mask limit line. This provides a margin for other causes of distortion, such as narrowband antenna operation.

**Figure 10:**
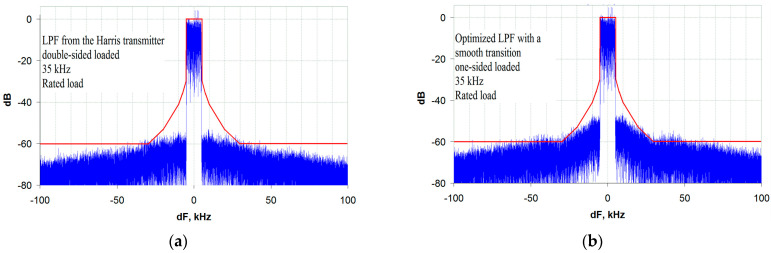
The transmitter output signal spectrum for: (**a**) a DSL LPF with an ST and a bandwidth of 35 kHz (3.5 bands of the transmitted signal, R_S_ = R_L_); (**b**) an optimized OSL LPF with an ST and a bandwidth of 35 kHz (3.5 bands of the transmitted signal, R_S_ = 0).

Finally, the third important conclusion obtained as a result of the analysis is that the envelope path bandwidth of about 3.5 bands of the transmitted signal is the minimum allowable (the limiting factor). In this case, out-of-band emissions caused by envelope spectrum reduction lie on the limit line of the mask, which provides no margin for the effects of other causes of distortion. As an illustration of this conclusion, Figure 10a,b show the spectra of the transmitter output signal for two types of LPF in the envelope path with R_S_ = R_L_ and R_S_ = 0.

### 3.3. One-Sided Loaded and Double-Sided Loaded LPF C0610

Consider next a class of LPFs with a standard Elliptic (Cauer) approximation of the sixth-order-type C0610. The structure of these filters coincides with the structure of the considered filters (see Figure 4) and differs only in the element values.

**Figure 11:**
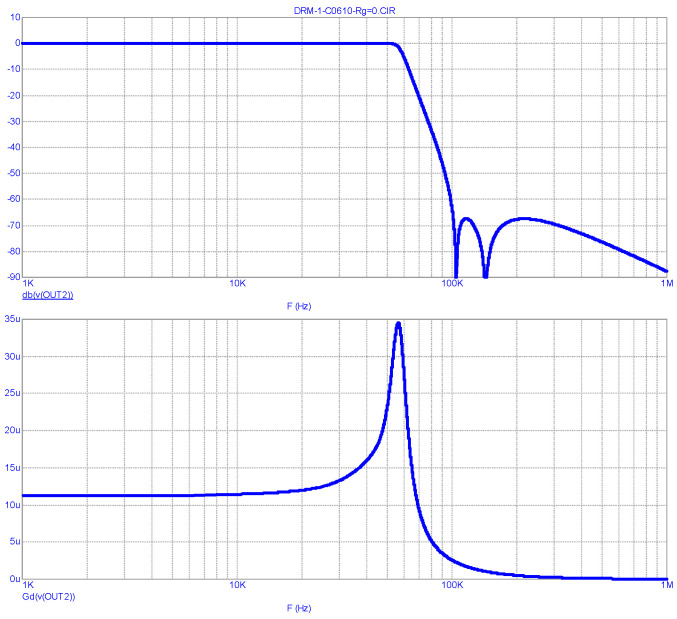
FR (**top**) and GD (**bottom**) of an OSL standard Elliptic (Cauer) C0610 LPF at R_S_ = 0. Cutoff frequency 58 kHz, rejection 67.5 dB.

**Figure 12:**
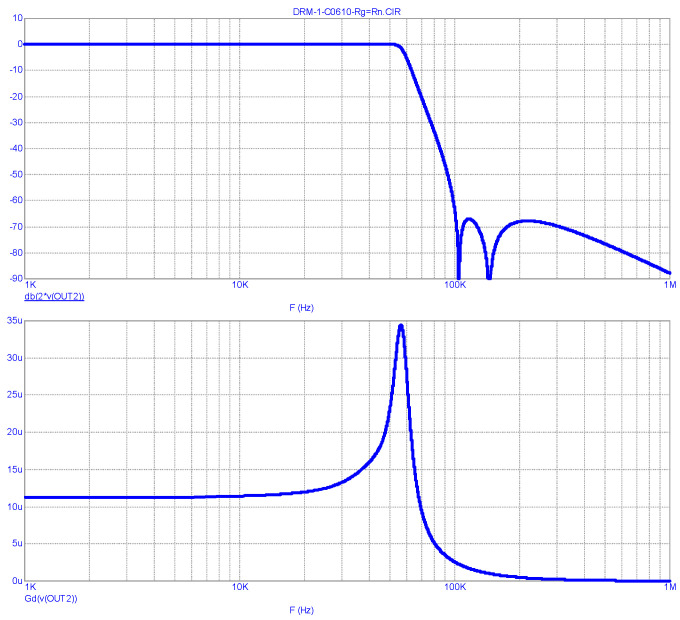
FR (**top**) and GD (**bottom**) of a DSL standard Elliptic (Cauer) C0610 LPF at R_S_ = R_L_. Cutoff frequency 58 kHz, rejection 67.5 dB.

**Figure 13:**
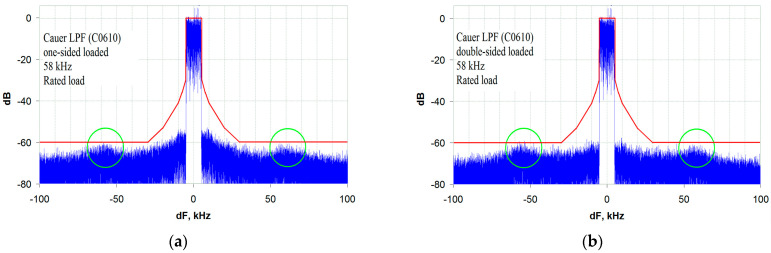
The transmitter output signal spectrum for standard Elliptic (Cauer) LPF (C0610) with a bandwidth of 58 kHz (≈6 bands of the transmitted signal): (**a**) OSL, R_S_ = 0; (**b**) DSL, R_S_ = R_L_.

The design is carried out according to the standard method described in [21]. The frequency characteristics of the two versions of the C0610 LPF—OSL and DSL—are shown in Figure 11 and Figure 12, respectively. It should be noted that these characteristics are completely identical for the corresponding modes of their excitation, R_S_ = 0 and R_S_ = R_L_. With a bandwidth of 66.2 kHz (at −3 dB), the GD is 10.2 µs and the stopband rejection is 78.5 dB. The transmitter output signal spectrum analysis results, obtained for these two versions of the LPF, are shown in Figure 13a,b at R_S_ = 0 and R_S_ = R_L_, respectively. Analyzing the given spectrograms, one should note the presence of local rises in the out-of-band emissions (marked with green ovals in Figure 13), which are separated from the central frequency by the LPF cutoff frequency.

**Figure 14:**
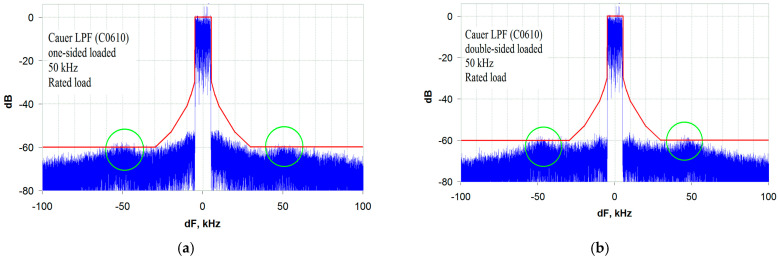
The transmitter output signal spectrum for a standard Elliptic (Cauer) LPF (C0610) with a bandwidth of 50 kHz (5 bands of the transmitted signal): (**a**) OSL, R_S_ = 0; (**b**) DSL, R_S_ = R_L_.

Out-of-band emission rises away from the transmitter center frequency at a distance equal to the LPF cutoff frequency have already been discussed. They severely limit the minimum allowable envelope path bandwidth when using standard filters without an ST. The minimum envelope path bandwidth for a standard LPF is five bands of the transmitted signal, as shown in the spectrograms in Figure 14a,b.

Thus, having considered four variants of the investigated envelope path filters (OSL and DSL with a smooth and sharp transition, respectively) possessing the same basic parameters (cutoff frequency and unwanted PWM clock frequency product suppression), we can draw the following conclusions:

- A sharp transition from the passband to the stopband in the envelope path LPF leads to an increase in out-of-band emissions that are separated from the transmitter center frequency by the LPF cutoff frequency.
- The minimum allowable bandwidth for the envelope path when using an Elliptic (Cauer) LPF with a standard approximation is at least five bands of the transmitted signal.
- The minimum allowable bandwidth for the envelope path when using an optimized LPF with an ST is at least 3.5 bands of the transmitted signal.
- When the transmitter is operating at a nominal load (broadband antenna), its linearity does not depend on the LPF type (OSL or DSL), provided that the signal source (modulator) output impedance is equal to that adopted when designing the filter.

## 4. Relationship between Antenna Bandwidth and Maximum VSWR at Signal Band Edges

Let us determine the relationship between the antenna bandwidth and the maximum VSWR at the boundaries of the signal band. To achieve this, in the AWR Microwave Office 2002 (MWO) program, antenna VSWR graphs are plotted for various bandwidths. In this case, the antenna is represented by a model in the form of a series LCR circuit, the parameters of which are calculated in the MWO program according to the formulas given below. The first two lines define the analysis parameters—antenna bandwidth B(ΔF) and central operating frequency $f_{0}$. Next, the antenna quality factor $Q_{a}$ is calculated, and for a given antenna resistance *Ra*, the characteristic impedance ρ of the series circuit is calculated.

The last two lines calculate the inductance and capacitance of the antenna series circuit:
Calculation expressionsMWO syntax$\begin{array}{l} \Delta F=205\;\mathrm{kHz}\\ f_{0}=150\;\mathrm{kHz}\\ Q_{a}=f_{0}/\Delta F\\ R_{a}=50\\ \rho =Q_{a}R_{a}\\ L_{a}=\rho /(2\pi f_{0})\\ C_{a}=1/(\rho 2\pi f_{0}) \end{array}$$\begin{array}{l} dF=205e3\\ f0=150000\\ Qa=(f0/dF)\\ Ra=50\\ RO=Qa*Ra\\ Pi=3.141592654\\ La=RO/(2*Pi*f0)\\ Ca=1/(RO*2*Pi*f0) \end{array}$

**Figure 15:**
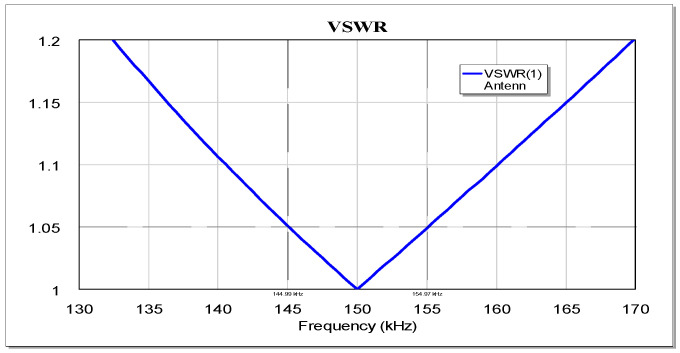
Frequency dependence of the VSWR of an antenna with a 205 kHz bandwidth.

As a result of the calculations carried out in the Microwave Office program, the dependence of the antenna VSWR on the frequency offset from the antenna center frequency is determined. As an example, Figure 15 shows the VSWR FR for a 205 kHz antenna bandwidth.

**Figure 16:**
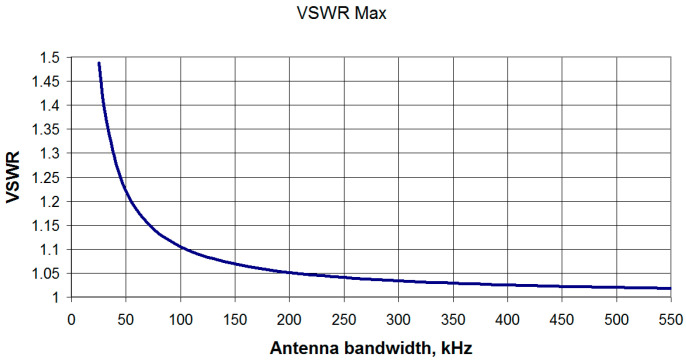
Dependence of the antenna circuit maximum VSWR at the signal band boundaries on the antenna bandwidth at a level of minus 3 dB.

From the graph (see Figure 15), the VSWR is determined at frequencies of ± 5 kHz from the center frequency—in this case equal to 1.05. Similar calculations are carried out for different antenna bandwidths, and on their basis a graph is constructed (Figure 16) that relates the antenna bandwidth and the VSWR value at the signal band boundaries (±5 kHz).

Note that the graph shown in Figure 16 is valid for any operating frequency, which follows from the analysis of the above formulas and is confirmed by the calculations carried out in the Microwave Office 2002 program. In addition, from this graph (Figure 16) one can determine the maximum VSWR for any signal band. Thus, for example, for a signal with a double bandwidth (±10 kHz), one would need to take a point corresponding to half of the antenna bandwidth and determine the VSWR at the signal band extreme frequencies. As an example, consider an antenna with a bandwidth of 205 kHz and a signal with a bandwidth of ±10 kHz. From the graph in Figure 16, we can determine the antenna VSWR for half the bandwidth of the antenna (102.5 kHz) and obtain a VSWR value = 1.1. To confirm the above, consider Figure 15 and verify that with a real antenna bandwidth of 205 kHz for a ±10 kHz signal bandwidth, the SWR is indeed 1.1.

The results obtained during the simulation require a fairly rigorous mathematical confirmation process, during which it is also possible to determine the limits of their applicability. The expression for the VSWR, according to its definition, is written as [24]

(1) $$VSWR=\frac{1+\left|\Gamma \right|}{1-\left|\Gamma \right|},$$

where $\left|\Gamma \right|$ is the modulus of the reflection coefficient.

The reflection coefficient is defined as [24]

(2) $$\Gamma =\frac{\left(Z_{L}-Z_{W}\right)}{\left(Z_{L}+Z_{W}\right)}.$$

In the case of an arbitrary load $Z_{L}=R_{L}+jX_{L}$ in the absence of attenuation in the line, the modulus of the reflection coefficient [24] is

(3) $$\left|\Gamma \right|=\sqrt{\frac{{\left(R_{L}-W\right)}^{2}+X_{L}^{2}}{{\left(R_{L}+W\right)}^{2}+X_{L}^{2}}}.$$

Since above and in [14] the antenna input impedance active component $R_{A}$ is considered constant in the signal frequency band (and equal to the feeder wave impedance W), Expression (3) takes the form

(4) $$\left|\Gamma \right|=\sqrt{\frac{X_{A}^{2}}{{\left(2R_{A}\right)}^{2}+X_{A}^{2}}}.$$

Let us write the expressions for the antenna input impedance reactive component (represented by an equivalent series circuit) at the boundaries of the useful signal frequency band F:

(5) $$\begin{array}{l} X_{A1}=2\pi (f+\frac{F}{2})L-1/(2\pi (f+\frac{F}{2})C);\\ X_{A2}=2\pi (f-\frac{F}{2})L-1/(2\pi (f-\frac{F}{2})C). \end{array}$$

Given that $Q=\frac{\rho }{R}=\frac{2\pi fL}{R}=1/(2\pi fCR)$, we express L and C as

(6) $$\begin{array}{l} L=\frac{QR}{2\pi f};\\ C=1/(2\pi fQR). \end{array}$$

Substituting (6) into (5), after transformations we obtain

(7) $$\begin{array}{l} X_{A1}=QR\left[\left(1+\frac{F}{2f}\right)-1/\left(1+\frac{F}{2f}\right)\right];\\ X_{A2}=QR\left[\left(1-\frac{F}{2f}\right)-1/\left(1-\frac{F}{2f}\right)\right]. \end{array}$$

Let us transform (7) into the form

$$\begin{array}{l} X_{A1}=QR\frac{{\left(1+\frac{F}{2f}\right)}^{2}-1}{\left(1+\frac{F}{2f}\right)}=QR\frac{1^{2}+\frac{F}{f}+{\left(\frac{F}{2f}\right)}^{2}-1}{\left(1+\frac{F}{2f}\right)};\\ X_{A2}=QR\frac{{\left(1-\frac{F}{2f}\right)}^{2}-1}{\left(1-\frac{F}{2f}\right)}=QR\frac{1^{2}-\frac{F}{f}+{\left(\frac{F}{2f}\right)}^{2}-1}{\left(1-\frac{F}{2f}\right)}, \end{array}$$

Neglecting the term of the second order of smallness ${\left(F/2f\right)}^{2}$, which leads to an error of no more than 2.5% at F/f≤0.1, we obtain

(8) $$\begin{array}{l} X_{A1}=QR\frac{F}{\left(f+\frac{F}{2}\right)};\\ X_{A2}=QR\frac{-F}{\left(f-\frac{F}{2}\right)}. \end{array}$$

Taking into account Q=f/B, where B is the antenna bandwidth at a level of minus 3 dB, substituting (8) into (4), we obtain

(9) $$\begin{array}{l} {\left|\Gamma \right|}_{1}=\frac{R_{A}\frac{f}{B}\frac{F}{\left(f+F/2\right)}}{\sqrt{4R_{A}^{2}+{\left(R_{A}\frac{f}{B}\frac{F}{\left(f+F/2\right)}\right)}^{2}}}=\frac{\frac{f}{B}\frac{F}{\left(f+F/2\right)}}{\sqrt{4+{\left(\frac{f}{B}\frac{F}{\left(f+F/2\right)}\right)}^{2}}};\\ {\left|\Gamma \right|}_{2}=\frac{R_{A}\frac{f}{B}\frac{F}{\left(f-F/2\right)}}{\sqrt{4R_{A}^{2}+{\left(R_{A}\frac{f}{B}\frac{-F}{\left(f-F/2\right)}\right)}^{2}}}=\frac{\frac{f}{B}\frac{F}{\left(f-F/2\right)}}{\sqrt{4+{\left(\frac{f}{B}\frac{-F}{\left(f-F/2\right)}\right)}^{2}}}. \end{array}$$

Assuming that at F/f≤0.1, the expression f/(f±F/2) with an error of no more than 5% can be considered equal to unity, Expression (9) can be represented as

(10) $${\left|\Gamma \right|}_{1,2}\approx \frac{F/B}{\sqrt{4+{\left(F/B\right)}^{2}}},$$

and, accordingly, the VSWR from (1) can be written as

(11) $$VSWR=\frac{\sqrt{4+{\left(F/B\right)}^{2}}+F/B}{\sqrt{4+{\left(F/B\right)}^{2}}-F/B}.$$

This expression confirms the independence of the VSWR value at the edges of the signal band from the value of the operating frequency obtained as a result of modeling. The dependence of the VSWR on the bandwidth calculated in accordance with (11) for the frequency band of the useful signal coincides with the simulation results shown in Figure 16.

**Figure 17:**
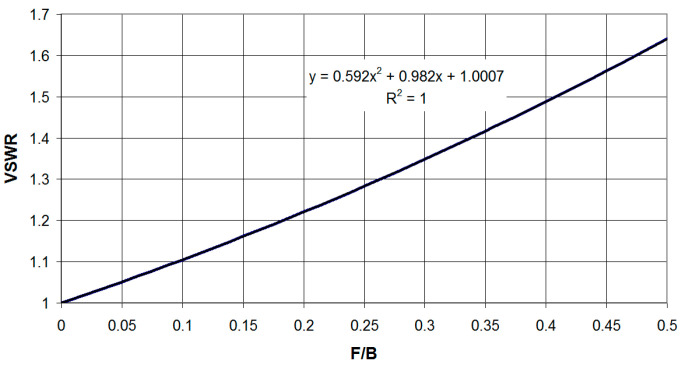
Dependence of the antenna circuit maximum VSWR at the boundaries of the useful signal bandwidth on the ratio to the antenna bandwidth.

The dependence of the VSWR on the F/B ratio is shown in Figure 17 and can be approximated on the interval F/B∈[0;0.5] by a quadratic function $VSWR=0.592{\left(F/B\right)}^{2}+0.982(F/B)+1.0007$ with $R^{2}=1$.

## 5. Analysis of the Transmitter’s Output Signal Spectral Characteristics during Its Operation with a Narrow-Band Antenna

Now let us proceed directly to identifying the dependence of the minimum required envelope path filter bandwidth on the antenna bandwidth. To achieve this, in the developed model [19], various antenna bandwidths are specified, and the minimum necessary envelope path LPF bandwidth at which the out-of-band emission levels do not exceed the allowable values is determined. The operating frequency is further taken to be 150 kHz, the most difficult for the implementation of the considered EER broadcast transmitters in the LF range.

### 5.1. Double-Sided Loaded LPF with a Smooth Transition

**Figure 18:**
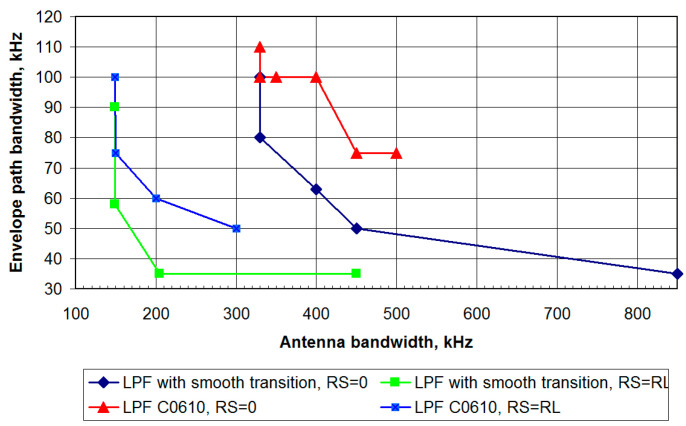
Dependence of the envelope path minimum required bandwidth on the antenna bandwidth.

Based on the analysis results, a plot of the envelope path minimum required bandwidth’s dependence on the antenna bandwidth is plotted. For a DSL LPF with an ST, the graph is shown in Figure 18 as the curve labeled “LPF with smooth transition, R_S_ = R_L_”.

**Figure 19:**
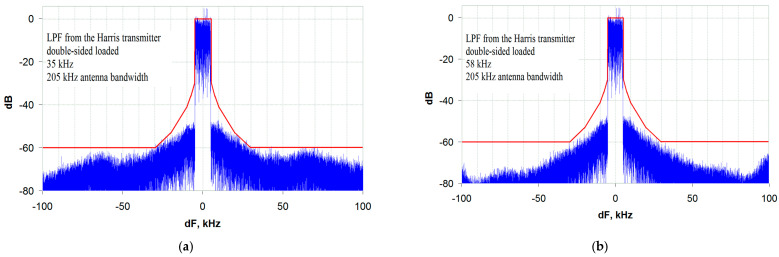
Transmitter output spectrum when operating on an antenna with a bandwidth of 205 kHz (20.5 bands of the transmitted signal) using a DSL LPF with an ST and a bandwidth of: (**a**) 35 kHz (3.5 bands of the transmitted signal); (**b**) 58 kHz (5.8 bands of the transmitted signal).

Analyzing the obtained dependence, we note the following. The horizontal line on the graph is determined by the minimum allowable bandwidth of the envelope path at which distortion is determined by the envelope spectrum limitation (3.5 bands of the transmitted signal). With an antenna bandwidth of more than 205 kHz (20.5 bands of the transmitted signal), the distortions caused by the limited bandwidth of the antenna are still not affected, which is confirmed by the spectrogram shown in Figure 19a.

In this case, distortions (out-of-band emissions) can still be reduced by expanding the envelope path bandwidth, which is confirmed by the spectrogram in Figure 19b obtained for the same antenna bandwidth but with an expanded envelope path bandwidth.

In a small range of antenna bandwidth from 205 kHz to 150 kHz, the minimum required envelope path bandwidth is linearly dependent on the antenna bandwidth. In this range of antenna bandwidths, the distortion (out-of-band emissions) arising from each of the causes of distortion—the envelope spectrum limitation and the NB antenna—is not yet at the spectral mask requirement limit. Therefore, both sources of distortion are acceptable, which together bring the out-of-band emissions to the maximum allowable value (up to the limiting mask).

**Figure 20:**
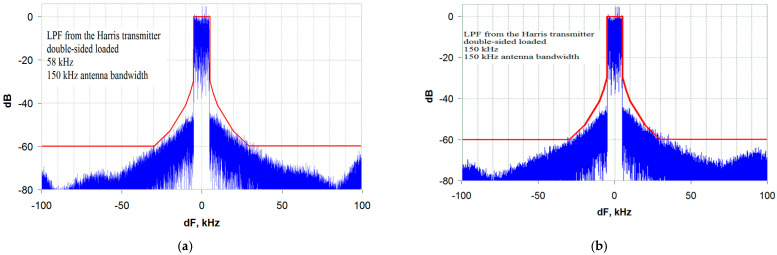
Transmitter output spectrum when operating on an antenna with a bandwidth of 150 kHz (15 bandwidths of the transmitted signal) using a DSL LPF with an ST and a bandwidth of: (**a**) 58 kHz (5.8 bandwidths of the transmitted signal); (**b**) 150 kHz (15 bandwidths of the transmitted signal).

With narrow antenna bandwidths (less than 150 kHz), the distortion caused by a narrowband antenna already exceeds the mask requirements and cannot be reduced by broadening the envelope path bandwidth. The distortions caused by the envelope spectrum limitation with an LF path bandwidth of more than 58 kHz are negligible and do not make a significant contribution to the overall level of distortions (out-of-band emissions), which is confirmed by the spectrograms in Figure 20a,b. It can be seen from the spectrograms that the out-of-band emission level does not decrease with an increasing envelope path bandwidth. This circumstance is reflected in the graph in Figure 18 with a vertical line that defines the minimum possible antenna bandwidth (150 kHz or 15 bandwidths of the transmitted signal) at which the EMC requirements are still met when using a DSL LPF with an ST.

Let us now consider a transmitter operating on an NB antenna using an OSL LPF with an ST.

### 5.2. One-Sided Loaded LPF with a Smooth Transition

As a result of a similar analysis of the transmitter operating with an OSL LPF on an NB antenna, the dependence of the minimum envelope path bandwidth on the antenna bandwidth is revealed, as shown in Figure 18 by the curve labeled “LPF with smooth transition, R_S_ = 0”.

**Figure 21:**
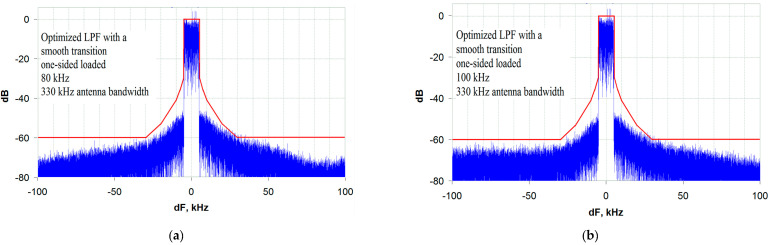
Transmitter output spectrum when operating on an antenna with a bandwidth of 330 kHz (33 bandwidths of the transmitted signal) using an OSL-optimized LPF with an ST and a bandwidth of: (**a**) 80 kHz (8 bandwidths of the transmitted signal); (**b**) 100 kHz (10 bandwidths of the transmitted signal).

Analyzing the dependence given in Figure 18, it should be noted that an OSL LPF with an ST possessing identical parameters is significantly inferior to a DSL LPF with an ST in terms of its ability to operate on an NB antenna. Thus, the minimum allowable antenna bandwidth is 330 kHz (33 bands of the transmitted signal), which is almost twice that in the case of using the DSL LPF discussed above. As an example, Figure 21 shows the spectrograms of the output signal with the minimum allowable antenna bandwidth of 330 kHz.

As follows from the spectrogram analysis in Figure 21, the minimum allowable antenna bandwidth for this type of LPF is 330 kHz, and the distortion (out-of-band emissions) does not decrease with an increasing envelope path bandwidth. This suggests that the maximum allowable distortion (out-of-band emissions) is completely determined by the NB antenna and cannot be reduced by expanding the envelope path bandwidth.

Otherwise, the behavior of the dependence in Figure 18 is similar to that of the dependence for a DSL LPF with an ST. Next, we consider the transmitter’s operation on an NB antenna with a standard Elliptic (Cauer) C0610 LPF in the envelope path.

### 5.3. One-Sided Loaded LPF C0610

First, the operation of the EER transmitter is studied when the filter is excited by a voltage source with R_S_ = 0. The corresponding graph of the dependence of the envelope path’s minimum required bandwidth on the antenna bandwidth is shown in Figure 18 by the curve labeled “LPF C0610, R_S_ = 0”.

Comparing the obtained dependence with the similar dependence for the optimized LPF with an ST (see Figure 18), it can be seen that the standard LPF is significantly inferior to the latter. Thus, we can conclude that it is undesirable to use standard filters when creating an EER transmitter envelope path.

This is due to the fact that the designers of standard filters did not pay attention to the nature of the FR immediately after the cutoff frequency. At the same time, as noted above, this region plays an important role in the convolution process of the envelope and the phase-modulated component of the transmitted signal. In the presence of a sharp break in the FR at the passband boundary, the energy of out-of-band oscillations arising from the limitation of the filter passband turns out to be concentrated in a narrow frequency band. This produces characteristic bursts on the signal spectrogram in frequency zones spaced above and below the central operating frequency at a distance equal to the LPF frequency cutoff.

### 5.4. Double-Sided Loaded LPF C0610

Next, the standard Elliptic (Cauer) filter is considered when working with a matched envelope signal source. The calculation results are shown in Figure 18 by the curve labeled “LPF C0610, R_S_ = R_L_”. A comparison of the obtained results with the case of the OSL filter confirms that the use of a modulator with a matched output impedance is also preferable, since it allows one to reduce the requirements for the antenna bandwidth and its VSWR.

## 6. Development of Recommendations for Envelope Path LPF Parameter Selection

Having considered and analyzed the options for implementing the envelope path LPF, we note that filters with an ST ensure the operation of the transmitter on antennas with a narrower bandwidth than standard LPF implementations.

Of the filters with an ST, the best is the DSL LPF, provided that it is matched at the input, i.e., the modulator output impedance is resistive and is equal to the LPF nominal load impedance. However, such a solution requires the special development of the modulator hardware.

For a transmitter operating on a resonant antenna with a limited bandwidth, the dependences of the envelope path LPF minimum required bandwidth on the antenna bandwidth and the SWR value at the signal band edges were revealed in Figure 18. The results obtained are shown in Table 1.

From the analysis and comparison of the obtained results, it follows that the use of a DSL LPF in the envelope path allows the transmitter to work on antennas with half the bandwidth or, in other words, reduces the requirements for the antenna SWR from 1.03 to 1.07. The use of filters with an ST allows one to reduce the minimum required LPF bandwidth by 20% compared to standard filters.

The most preferable is the use of a sixth-order DSL LPF with an ST, which ensures that the transmitter can operate on an antenna with SWR = 1.07 at the edges of the transmitted signal band and a minimum LPF bandwidth equal to 58 kHz.

## 7. Conclusions

Studies of the out-of-band emissions of EER transmitters were carried out using four envelope path LPF configurations (OSL and DSL with a smooth and sharp transition, respectively) for both the nominal load (broadband antenna) and resonant antennas with a limited bandwidth.

When the transmitter is operating at a rated load (broadband antenna):

- The minimum allowable bandwidth of the envelope path when using an optimized LPF with an ST is at least 3.5 bands of the transmitted signal.
- When the transmitter is operating at a nominal load (broadband antenna), its linearity does not depend on the LPF type (OSL or DSL).

For the case of transmitter operation on a resonant antenna with a limited bandwidth:

- It is shown that the use of a DSL LPF in the envelope path allows the transmitter to work on antennas with half the bandwidth or, in other words, to ease the antenna VSWR requirements from 1.03 to 1.07.
- The most preferable is the use of a sixth-order DSL LPF with an ST, which ensures that the transmitter can operate on an antenna with VSWR = 1.07 at the edges of the transmitted signal band and a minimum LPF bandwidth equal to 5.8 bands of the amplified signal.

The use of the proposed modulator configuration featuring a double-sided loaded LPF with a smooth transition allows the transmitter to operate on an antenna with VSWR = 1.07 at the edges of the transmitted signal band and a minimum LPF bandwidth equal to 5.8 bands of the amplified signal. This significantly expands the capabilities of its application and allows one to reduce the PWM clock frequency and increase the efficiency.

Experimental studies are quite complex and are expected as a future research direction.

## Figures and Tables

**Table 1:** Calculation results for the dependence of the envelope path LPF minimum required bandwidth on the antenna bandwidth and the SWR value at the signal band edges.

Envelope Path LPF Implementation Option	Minimum Antenna Bandwidth, Multiplied by Signal Bands	Minimum Required LPF Bandwidth, Multiplied by Signal Bands	Maximum Allowable Antenna VSWR at the Signal Band Edges
***LPF with standard frequency response***			
OSL standard 6th-order LPF	33	10	1.03
DSL standard 6th-order LPF	15	7.5	1.07
***LPF with a smooth transition***			
OSL 6th-order LPF with a ST	33	8	1.03
DSL 6th-order LPF with a ST	15	5.8	1.07

## Data Availability

Data are contained within the article.
